# Imatinib and dasatinib as salvage therapy for sclerotic chronic graft-vs-host disease

**DOI:** 10.3325/cmj.2016.57.247

**Published:** 2016-06

**Authors:** Isabel Sánchez-Ortega, Rocío Parody, Octavio Servitje, Cristina Muniesa, Montserrat Arnan, Beatriz Patińo, Anna Sureda, Rafael F. Duarte

**Affiliations:** 1 Department of Clinical Hematology, Catalan Institute of Oncology, Hospital Duran i Reynals, Barcelona, Spain; 2 Department of Dermatology, Hospital Universitari de Bellvitge, Barcelona, Spain; 3 Department of Clinical Hematology, Hospital Universitario Puerta de Hierro, Madrid, Spain

## Abstract

**Aim:**

To assess the toxicity, tolerance, steroid-sparing capacity, effectiveness, and response rate to imatinib and dasatinib for the treatment of severe sclerotic chronic graft-vs-host disease (scGVHD).

**Methods:**

This retrospective study analyzed 8 consecutive patients with severe refractory scGVHD who received salvage therapy with imatinib. Patients intolerant and/or refractory to imatinib received dasatinib treatment.

**Results:**

7 patients discontinued imatinib treatment (1 achieved complete response, 5 were resistant and/or intolerant, and 1 developed grade IV neutropenia) and 1 patient achieved prolonged partial response, but died due to an infectious complication while on treatment. 5 patients started dasatinib treatment (3 achieved partial responses and discontinued dasatinib, 1 achieved a durable partial response, but died due to a consecutive rapid pulmonary cGVHD progression and 1 with stable disease discontinued treatment due to gastroenteric intolerance). The response rate (partial and/or complete responses) for severe scGVHD was 25% for imatinib and 60% for dasatinib.

**Conclusion:**

In our series, dasatinib was better tolerated, safer, steroid-sparing, and had a low incidence of infectious complications, which suggests that it may be a more effective therapeutic alternative for patients with refractory scGVHD than imatinib. Treatment of scGVHD with effective antifibrotic drugs such as TKI, which block the kinase fibrotic pathway, may be a safe and effective therapeutic option, but further studies are needed to confirm our findings.

Chronic graft-vs-host disease (cGHVD) is the major cause of late nonrelapse morbidity after allogeneic blood and marrow transplantation (1,2), occurring in approximately 50% of long-term survivors (3,4). Sclerotic chronic graft-vs-host disease (scGVHD) is one of the most severe forms of the disease, involved in the formation of a wide spectrum of fibrotic entities, in which the common cause of end-organ dysfunction is the excessive production of extracellular matrix by activated myofibroblasts (5). Inamoto et al showed the incidence of scGVHD after three years of initial systemic treatment for cGVHD to be 20% (3); other studies have reported rates ranging from 8% after two years of allogeneic transplant to 15% after five years in patients with cGVHD (4,6).

ScGVHD has limited and disappointing treatment options and is associated with considerable functional disability and morbidity, impaired quality of life, and prolonged pharmacological immunosuppression (3), leading to an additional increased risk of infections and other late complications.

Imatinib mesylate is a first generation tyrosine kinase inhibitor (TKI), successfully used in patients with bcr-abl positive leukemias (7,8), which inhibits profibrotic cytokines, transforming growth factor-β (TGF-β), and platelet-derived growth factor receptor (PDGFR) signaling pathways in addition to c-Abl (5). It has been shown to decrease fibrosis in preclinical models (9), phase II trials, and small cohorts of cGVHD patients (10-15).

Dasatinib is a second-generation TKI with a greater inhibitory potency, an improved toxicity profile, and proven clinical efficacy in the treatment of chronic myeloid leukemia patients refractory or intolerant to imatinib (16). It inhibits sarcoma-tyrosine (Src) kinases, which regulate c-Abl and are activated by TGF-β and PDGF (5), play a central role in the development of experimental dermal fibrosis (17), and effectively inhibit the synthesis of extracellular matrix in both *in vitro* and *in vivo* models (18). Moreover, dasatinib modulates myofibroblast differentiation through Src pathway, which makes it a potential therapeutic option for the treatment of fibrotic diseases (19).

Owing to their antifibrotic effects by blocking kinase fibrotic targets and their signaling pathways, we hypothesized that imatinib and dasatinib may be effective therapeutic alternatives for patients with scGVHD. We have already reported in a small series of patients the first direct clinical evidence suggesting that dasatinib may be a safe and effective therapeutic option for patients with severe scGVHD refractory to corticosteroids and resistant or intolerant to imatinib (20). Here, in a larger series of patients with longer follow-up, we evaluated the toxicity, tolerance, steroid-sparing capacity, effectiveness, and response rate to imatinib and dasatinib for the treatment of severe scGVHD.

## Patients and methods

We retrospectively described a series of 8 consecutive patients with severe scGVHD in whom at least two previous immunosuppressive treatment lines failed (Table 1) and who went on to receive salvage therapy with imatinib. Patients were treated at the Catalan Institute of Oncology, Barcelona, between January 2009 and December 2015. 5 patients were intolerant and/or refractory to imatinib and received dasatinib treatment. The median age at allogeneic transplantation was 53 (range, 27-67) years, the median time from allogeneic transplant was 23 months (range, 19-108), and the median time from scGVHD diagnosis was 18.5 months (range, 11-26). 7 patients presented with *de novo* chronic GVHD (87.5%). Patients’, transplant, and cGVHD characteristics are summarized in Table 1.

**Table 1:** Patients’, transplant, and graft-vs-host disease (GVHD) characteristics*

Patient #	1	2	3	4	5	6	7	8
**Sex**	Female	Male	Female	Male	Male	Male	Male	Female
**Age at SCT**	58	28	27	57	67	60	30	50
**Disease status at SCT**	1st CR AML	1st CR AML	1st CR AML	2nd PR, AML	1st CR AML	2nd CR, MM	1st uCR HL^‡^	1st CR AML
**Conditioning regimen**	RIC (Flu-Bu)/ ATG, CyA^†^	MAC (Cy-TBI)	MAC (Cy-TBI)	MAC (FLAG-Ida, Mel)	RIC (Flu-Bu)	RIC (Flu-Bu)	MAC (Bu-Cy)	MAC (Cy-TBI)
**GVHD prophylaxis**	CyA, MTX	CyA, MTX	CyA, MTX, ATG	CyA, MMF	CyA, MTX	CyA, MTX	CyA, MTX	CyA, MTX
**Donor relation; matches**	Related; 10/10	Related; 10/10	Unrelated; 10/10	Related; 10/10	Related; 10/10	Related; 10/10	Related; 10/10	Related; 10/10
**Stem cell source**	peripheral blood	peripheral blood	peripheral blood	peripheral blood	peripheral blood	peripheral blood	peripheral blood	peripheral blood
**Allogeneic SCT**	2007, September	2007, April	2008, January	2009, January	2008, March	2000, November	2010, April	2012, December
**Acute GVHD**	No	Grade III. Cutaneous Response to CS	No	No	No	No	No	No
**cGVHD onset, m after SCT**	*De novo*, 9	Quiescent, 5	*De novo*, 10	*De novo*, 16	*De novo*, 22	*De novo*, 22	*De novo*, 10	*De novo*, 7
**IS before TKI**	CyA 3mg/Kg/d, MMF 1gr/8h, CS	CyA 3mg/Kg/d, MMF 1gr/8h, CS, PUVA (10 sessions)	CyA 3mg/Kg/d, MMF 1gr/8h, CS	CyA 3mg/Kg/d, MMF 1gr/8h, CS	CyA 3mg/Kg/d, MMF 1gr/8h, CS	CsA 3mg/Kg/d, MMF 1gr/8h, CS	CyA 3mg/Kg/d, MMF 1gr/8h, CS	CyA 3mg/Kg/d, MMF 1gr/8h, Sirolimus 1mg/12h, PUVA, CS

*AML – acute myeloid leukemia; ATG – antithymocyte globulin; Bu – busulfan; CR – complete remission; CS – corticosteroids; Cy – cyclophosphamide; CyA – cyclosporine A; FLAG-Ida – fludarabine, cytarabine, idarubucin, Flu – fludarabine; HL – Hodgkin lymphoma; IS – immunosuppression; m – months; MAC – myeloablative conditioning; MEL – melphalan; MM – multiple myeloma; MMF – mofetil mycophenolate; MTX – methrotexate; PR – partial remission; PUVA – Psolaren-UV-A therapy; RIC – reduced intensity conditioning; SCT – stem cell transplantation; TBI – total body irradiation; TKI – tyrosine kinase inhibitors.

†Patient #1 required a second donor infusion (7.6x10^6^/Kg CD34+ previous 1mg/Kg ATG on days -2 to -1 and CyA) on day +64 because of primary graft failure.

‡Patient #7 required three chemotherapy treatment lines (ABVD: doxorubicin, bleomycin, vinblastine, dacarbazine; ESHAP: etoposide, cisplatin, cytarabine, methylprednisolone, and COPP: cyclophosphamide, vincristine, procarbazine, prednisone) to achieve the first unconfirmed complete remission.

The study was approved by the Ethics Committee of Catalan Institute of Oncology, Duran i Reynals Hospital, Barcelona and informed consent for off-label use of the drugs was given by all patients. Imatinib was started at a dose of 100 mg daily and in the absence of severe toxicity or intolerance escalated to 400 mg daily within 8 weeks. Dasatinib was started at a dose of 50 mg daily and in the absence of severe toxicities the dose was escalated to 100 mg daily within 8 weeks. Adverse events were graded according to the Common Terminology Criteria for Adverse Events, Version 4.0. (*http://ctep.cancer.gov/protocolDevelopment/electronic_applications/ctc.htm#ctc_40*). Supportive care, antimicrobial prophylaxis, and monitoring followed our standard protocols in keeping with international guidelines (21). Diagnoses other than scGVHD, including infections and drug reactions, were previously excluded and sclerodermatous features were histologically confirmed by skin biopsy (data not shown). Diagnosis and staging of scGVHD were performed according to the National Institutes of Health Consensus Conference on cGHVD (22), but reassessed and graded again according to the updated 2014 criteria (23) for the purpose of this study. Before the start of first and second generation TKI treatment, patients’ skin score, joint mobility, range of motion, global severity score, and Karnofsky Performance Status were determined, and any other possible site involvement was evaluated and graded. The chronic GVHD organ specific severity scores and response to TKI at every affected site were graded according to the National Institutes of Health Consensus Development Project on cGVHD (24) and evaluated at three monthly intervals from the start of treatment.

## Results

Before the start of TKI treatment, all 8 patients had severe chronic GVHD (23). Global and organ-specific severity scores at the start of TKI treatment (PRE) and at the time of withdrawal or death (POST) are shown in Table 2. Severe scoring was attributable to multiple severe organ involvement in 6 patients and single skin involvement in 2 patients. All 8 patients exhibited severe skin involvement with sclerotic features including deep tissue sclerosis and hidebound lesions unable to pinch, 2 patients had severe ulcerations, and 3 generalized pruritus. Additional severe cGVHD targets included joints and fascia with restricted range of motion (n = 4), the lung (n = 3), eyes (n = 2), mouth (n = 1), and gastrointestinal tract (n = 1). Additional cGVHD targets with mild or moderate severity are also summarized in Table 2.

**Table 2:** TKI treatment and scGVHD assessment before the start of TKI treatment (PRE) and at withdrawal or death (POST) according to NIH updated criteria (24)

Patient #	1		2		3		4		5		6		7		8	
**Main cGvHD targets**	Skin, lung, joints		Skin, joints, eyes, mouth		Skin, joints		Skin, joints		Skin, joints		Skin, lung, gi tract, mouth, eyes		Skin, joints		Skin, joints, mouth, eyes, lung, gi tract	
**Imatinib withdrawal,** m	Yes. Intolerant (gi) and resistant, 8		Yes. Intolerant (gi) and resistant, 3		Yes. Refractoriness (progressive scGVHD), 4		Yes. Grade IV neutropenia, 0.5		Yes. Complete remission, 49		No. Dead, partial response, 35		Yes. Intolerant (gi), 1		Yes. Intolerant (gi), 3	
**Dasatinib withdrawal,** m	No, Dead, progression 28		Yes, Partial response 57		Yes, Partial response 75		NA		NA		NA		Yes, Partial response 26		Yes. Intolerant (gi) 2.5	
**Time to IS withdrawal** (from TKI initiation), m	No. cGVHD progression		No. On low-doses MMF		68		30		34		No. Dead in partial response		23		No. On sirolimus	
**Percent CS reduction**	90% / 0%^†^		100%		100%		NA		100%		100%		100%		NA (refractoriness and discontinued)	
**TKI initiation**, date	Dasatinib, July/09		Dasatinib, December/09		Dasatinib, October/09		Imatinib, August/10		Imatinib, February/10		Imatinib, January/09		Dasatinib, March/12		Dasatinib, October/15	
**Time from SCT**, m	22		32		21		19		23		98^‡^		23		34	
**Time from scGVHD onset**, m	13		26		11		17		20		24		13		21	
**Time dasatinib initiation from imatinib**, m	8		3		4		NA		NA		NA		1		6	
**Overall GVHD severity, m**	**PRE**	**POST** **(28)**	**PRE**	**POST** **(57)**	**PRE**	**POST (75)**	**PRE**	**POST (0.5)**	**PRE**	**POST (49)**	**PRE**	**POST** **(35)**	**PRE**	**POST (26)**	**PRE**	**POST (2.5)**
**cGVHD overall score**	Severe (multiple organ)	Severe	Severe (multiple organ)	Moderate	Severe (multiple organ)	Moderate	Severe (Skin)	Severe	Severe (Skin)	No GVHD	Severe (multiple organ)	Moderate	Severe (multipleorgan)	Moderate	Severe (multipleorgan)	Severe
**Performance Status** Zubrod (Karnofsky)	3 (50%)	3 (50%)	2 (60%)	0 (100%)	2 (60%)	0 (100%)	1 (80%)	1 (80%)	2 (70%)	0 (100%)	2 (60%)	1 (90%)	2 (60%)	0 (100%)	3 (50%)	2 (60%)
**Joints and fascia** **P-ROM**	2	1	3	0	3	0	2	2	2	0	0	0	3	0	3	3
shoulder	3	4	4	7	5	7	7	7	5	7			4	7	3	3
elbow	4	5	5	7	5	7	5	5	5	7			5	7	4	4
wrist/finger	6	6	5	7	2	7	3	3	5	7			5	7	2	2
ankle	4	4	4	4	1	4	4	4	4	4			3	4	2	2
**Skin;** features score	3	3	3	2	3	2	3	3	3	0	3	2	3	2	3	3
score % BSA	2	2	2	1	3	1	2	2	2	0	2	2	2	1	2	2
deep sclerosis	Yes	Yes	Yes		Yes		Yes	Yes	Yes		Yes		Yes		Yes	Yes
hidebound	Yes	Yes	Yes		Yes		Yes	Yes	Yes		Yes		Yes		Yes	Yes
superficial sclerosis				Yes		Yes						Yes		Yes		
hyperpigmentation					Yes		Yes	Yes							Yes	Yes
hypopigmentation			Yes	Yes			Yes	Yes			Yes	Yes				
lichen planus-like															Yes	Yes
poikiloderma							Yes	Yes			Yes	Yes			Yes	Yes
pruritus			Yes		Yes				Yes							
**Hair**	Yes	Yes	Yes	Yes							Yes	Yes				
**Nails**	Yes	Yes	Yes	Yes							Yes	Yes				
**Ulceration**	Yes		Yes								Yes	Yes				
**Lung;** Symptom score Lung score (%FEV_1_)	3 3	3 3	0	0	0	0	0	0	0	0	2 2	1 1	0	0	2 1	2 1
**Mouth**; NIH score	0	0	2	0	0	0	0	0	0	0	2	1	0	0	3	3
**Genital tract** score	0	0	0	0	0	0	1	1	0	0	0	0	0	0	0	0
**Gastrointestinal** tract	0	0	0	0	0	0	0	0	1	0	3	1	0	0	2	2
**Eyes**	0	0	3	0	0	0	0	0	0	0	3	1	0	0	2	2
**Status at the last follow-up**	Dead		Alive		Alive		Alive		Alive		Dead		Alive		Alive	
**Date**	2011, November		2015, November		2015, November		2015, October		2015, October		2011, December		2015, December		2015, December	
**Primary disease status**	Complete remission		Complete remission		Complete remission		Complete remission		Complete remission		Complete remission		Complete remission		Complete remission	
**Months after SCT**	50		95		103		81		92		133		68		36	

*BSA – body surface area; CS – corticosteroids; gi – gastrointestinal; FEV1 – forced expiratory volume in 1 second; IS – immunosuppression; m – months; NA – not applicable; PRE – National Institutes of Health (NIH) scores before the start of TKI treatment; POST – NIH scores at withdrawal of TKI treatment or death; P-ROM – photographic range of motion; scGVHD – sclerotic chronic graft-vs-host disease, SCT – stem cell transplantation; TKI – tyrosine kinase inhibitors.

†Patient #1 achieved a durable partial response with 90% reduction of her initial corticosteroid dose with a consecutive severe cGVHD flare; corticosteroid treatment was then restarted, but the patient died 28 mo after starting dasatinib treatment due to rapid pulmonary cGVHD progression.

‡In patient #6 immunosuppression was restarted in January 2007.

Patient #5 was diagnosed with severe cutaneous scGVHD, moderate joints and fascia and mild gastrointestinal involvement 23 months after allogeneic transplantation. He started imatinib treatment with very good tolerance and no adverse events. At the first response assessment, three months after the start of imatinib, his sclerotic features started improving and by six months he had achieved partial response with objective improvement in erythema due to fasciitis, joint stiffness, and photographic range of motion. His sclerotic features continued to progressively improve and additional immunosuppressive treatment was stopped 34 months later. After 49 months on imatinib, he achieved complete remission and treatment was withdrawn. At the last follow-up, 22 months after imatinib withdrawal, he remained off immunosuppression and in complete scGVHD and primary disease remission.

5 patients (62.5%) were resistant and/or developed gastrointestinal intolerance to imatinib after a median of 3 (range, 1-8) months, which required treatment discontinuation, and 1 case (patient #4) developed grade IV neutropenia two weeks after the start of treatment, which resolved after imatinib withdrawal. Finally, there was 1 non-relapse death (patient #6) 35 months after the start of imatinib treatment due to an acute pulmonary complication and a subsequent septic shock of unknown origin. At the time of death, he remained on a stable partial response of scGVHD with NIH scores improvement in all target organs (the skin, lung, mouth, gastrointestinal tract, and eyes) and his primary disease was in complete remission 133 months after transplantation (Table 2). The response rate (partial and/or complete responses) (24) for severe scGVHD in our series was 25% for imatinib (2/8).

5 patients resistant or intolerant to imatinib started dasatinib treatment. 3 of them had already experienced disease response at the first response assessment three months after the start of dasatinib (patients #2, #3, and #7) as assessed by a decrease in NIH skin, joints and fascia, mouth, and eyes scores, increase in photographic range of motion scores, improvement in Karnofsky Performance Status, and resolution of skin ulcers. Dasatinib was very well tolerated, with no adverse events. At the consecutive quarterly response assessments, scGVHD continued improving, leading to a discontinuation of dasatinib treatment in all three cases in a median of 57 months (range, 26-75) after the start of treatment. These patients had already discontinued corticosteroids and all additional immunosuppressive treatments, except the patient #2, who continued to receive low doses of mofetil mycophenolate. At the last follow-up, all three patients were alive and continued to have good scGVHD response with no requirement for additional immunosuppressive treatment and their primary disease in complete remission for a median of 95 months (range, 68-103) after transplantation. One patient (patient #1) achieved partial response three months after the start of dasatinib treatment with improvement in sclerotic features, skin thickness, joint mobility, and pulmonary involvement. By 12 months of dasatinib treatment she had become oxygen-independent at rest and her initial corticosteroid dose had been reduced by more than 90% (20). However, two months later her pulmonary symptoms started worsening, with progressive shortness of breath and oxygen requirement. Corticosteroid treatment was restarted, but she died 28 months after the start of dasatinib treatment due to rapid pulmonary cGVHD progression. Finally, patient #8 previously intolerant to imatinib, continued to have a stable disease after 2.5 months on dasatinib, showing objective improvement in body surface area involved by deep sclerotic features although not measurable by NIH skin score. However, dasatinib had to be discontinued due to grade 2 nausea and vomiting. At the time of dasatinib withdrawal and compared to the previous month, her cGVHD symptoms were a little better (+1) and the cGVHD severity grade had decreased from 8 to 6 despite the fact that both the clinician and the patient reported that perceived severity of sclerosis did not decrease, being thus far, severe (24) (data not shown). At the last follow-up, 5 patients had been on dasatinib for a median of 28 months (range, 2.5-75), with no grade 3-4 adverse events and a very good tolerance. Only 1 patient (#8) required early treatment discontinuation due to grade 2 gastrointestinal intolerance. We observed 3 partial responses to dasatinib (60%, 3/5) according to NIH (24) and 1 stable disease (20%). An additional patient (20%) showed partial response during 27 months, with a consecutive severe cGVHD flare and death.

There were 2 infectious complications (pneumococcal meningitis in patient #3 while on dasatinib with good response to antibiotic treatment and septic shock and death in patient #6 while on imatinib), in 5 patients corticosteroid treatment could be withdrawn, and all patients had a complete response of their primary disease at the last follow-up (Table 2).

Overall, 5 patients (62.5%, 2 on imatinib and 3 on dasatinib) achieved partial or complete responses of scGVHD (24). The response rate for imatinib was 25% (2/8; 1 patient was able to discontinue TKI and all additional immunosuppressive treatments) and 60% for dasatinib (3/5; 3 patients were able to discontinue TKI and 2 all additional immunosuppressive treatments). Overall, 6 patients were alive at the last follow-up. a median of 86.5 months (range 36-103) after transplantation.

## Discussion

In our series, the response rate (partial and/or complete responses) for severe scGVHD was 60% for dasatinib and 25% for imatinib. The most common adverse event related to imatinib that required treatment discontinuation was gastrointestinal intolerance. Olivieri et al (14) also reported toxicities occurring during the first three months of treatment. Baird et al (15) described the poor gastrointestinal tolerance of imatinib when administered at the doses of 200 mg or greater. Therefore, the high gastrointestinal toxicity associated to imatinib in our study could have been related to the higher dose administered.

On the other hand, dasatinib was very well tolerated in our series, with only a single case of grade 2 gastrointestinal intolerance. At scGVHD diagnosis, 6 patients had severe scoring attributable to multiple severe organ involvement. 3 of them had severe joint and range of motion limitation and severe cutaneous sclerotic features. According to NIH severity scores changes, the joint and fascia and the photographic range of motion scores showed complete response to dasatinib (24), however their skin NIH grading only decreased to a score of 2 on the NIH 0-3 point-scale (23), as residual superficial sclerotic features were still present at the last-follow-up. Thus, the global severity score just decreased from severe to moderate but patients’ range of motion normalized and their sclerosis, quality of life, and performance status significantly improved, enabling withdrawal of additional immunosuppressive treatments.

Our data suggest that dasatinib may be considerably better tolerated and a more effective therapeutic option than imatinib for patients with refractory scGVHD. Dasatinib was safer than imatinib, steroid-sparing, and had a low incidence of infectious complications. However, the administration of high doses of imatinib could have in part accounted for the high gastrointestinal toxicity. Therefore, lower doses of imatinib may be recommended.

ScGVHD is often refractory to standard immunosuppressive treatments and remains a significant problem for many long-term survivors. First and second generation TKI target fibrotic signaling pathways that cause organ fibrosis, representing a potential valuable salvage therapy for patients with refractory scGVHD. Advantages of TKI treatment include a well-established safety profile, an easy management of drug interactions, and an oral route of administration, a feature considerably important for patients’ and their quality of life. Treatment of scGVHD with antifibrotic drugs that block the kinase fibrotic pathway may be a safe and effective therapeutic option. However, the small number of patients in our series is a significant limitation, and many additional issues remain unexplained, such as the pathophysiology of scGVHD and the factors predicting the successful use of TKI in this setting. This is emphasized in an interesting case report by Pulanic et al (25), where an allogeneic stem cell transplant recipient developed severe scGVHD while on dasatinib treatment due to persistence of residual chronic myeloid leukemia after transplant. Further larger studies and clinical trials are warranted to determine appropriate patient selection, optimal doses, and duration of therapy for immunosuppressed patients.
